# Locally Injectable Hydrogels for Tumor Immunotherapy

**DOI:** 10.3390/gels7040224

**Published:** 2021-11-22

**Authors:** Xinyi Zhang, Xiaonan Guo, Yan Wu, Jie Gao

**Affiliations:** 1 Institute of Translational Medicine, Shanghai University, Shanghai 200444, China; zhangxy_913@shu.edu.cn; 2 Department of Hemangioma and Vascular Malformation, People’s Hospital of Zhengzhou University, Zhengzhou 450003, China; xiaonan_g@126.com; 3 Department of Hemangioma and Vascular Malformation, Henan Provincial People’s Hospital, Zhengzhou 450003, China; 4 Department of Biomaterial, College of Life Sciences, Mudanjiang Medical University, Mudanjiang 157011, China

**Keywords:** injectable hydrogel, immunotherapy, cancer, local administration, combined therapy

## Abstract

Hydrogel-based local delivery systems provide a good delivery platform for cancer immunotherapy. Injectable hydrogels can directly deliver antitumor drugs to the tumor site to reduce systemic toxicity and achieve low-dose amplification immunotherapy. Therefore, it may overcome the problems of low drug utilization rate and the systemic side effects in cancer immunotherapy through systemic immune drugs, and it provides simple operation and little invasion at the same time. This study aimed to review the research progress of injectable hydrogels in tumor immunotherapy in recent years. Moreover, the local delivery of multiple drugs using injectable hydrogels in tumors is introduced to achieve single immunotherapy, combined chemo-immunotherapy, combined radio-immunotherapy, and photo-immunotherapy. Finally, the application of hydrogels in tumor immunotherapy is summarized, and the challenges and prospects for injectable hydrogels in tumor immunotherapy are proposed.

## 1. Introduction

Cancer remains one of the greatest threats to human health even with the ongoing studies for its treatment. Three traditional treatments, namely surgery, radiotherapy, and chemotherapy, remain the most common treatments [1]. However, surgery may be accompanied by wound infection and incomplete resection. Chemotherapy drugs are able to kill tumor cells but damage normal cells simultaneously and cause the corresponding side effects [2]. Meanwhile, radiotherapy targets and kills tumor tissues using ionizing radiation, but normal tissues may be “accidentally injured”, leading to toxicity [3]. Moreover, only a small number of patients have received effective chemotherapy or radiotherapy treatments [2]. Most importantly, neither chemotherapy, radiotherapy, nor surgical resection can effectively inhibit cancer metastasis. Cancer recurrence and metastasis after several years are major problems leading to treatment failure or even death [4].

Immunotherapy has received extensive attention in recent decades and has made significant progress in cancer treatment. This therapy combats cancer by activating and utilizing the patient’s own immune system to fight the malignant cells. In normal organisms, the immune system recognizes and destroys the malignant cells. However, tumor cells release immunosuppressive molecules that inhibit other immune cells [5]. The immunosuppressive tumor microenvironment (TME) can lead to an insufficient infiltration of effector lymphocytes and a depletion of function, greatly weakening the antitumor immune effect, prompting the tumor to become “cold” and making the tumor immunotherapy far less effective than expected [6]. Therefore, the design of a reasonable treatment strategy to effectively turn “cold” tumors into “hot” tumors using active effector lymphocytes is the key to improve the effect of antitumor immunotherapy [7]. An appropriate immunotherapy enables the immune system to regain an anticancer role in the TME, thereby controlling or even eliminating cancer cells. However, the overall efficiency of immunotherapy in clinical treatment is insufficient [5]. It takes a cycle of systemic administration to activate the immune system, involving a variety of immune factors and processes. Similar to dominoes, no ring of interruptions can advance [8]. Therefore, local immunotherapy provides a promising method for drug release at target sites, which minimizes systemic toxicity and improves treatment efficiency.

**Figure 1:**
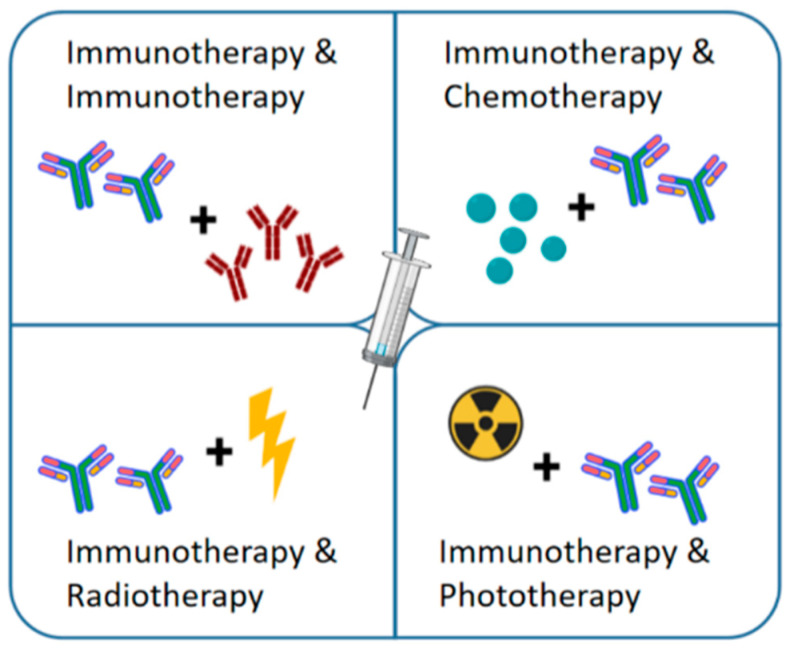
Diagram showing injectable hydrogels as unique platforms for local immunotherapy and combined therapy in tumors. Combined treatment strategies include immunotherapy, chemoimmunotherapy, radioimmunotherapy, and photoimmunotherapy.

To utilize the promising clinical applications of immunotherapy, researchers are constantly exploring biological scaffold materials as a delivery system for the local release of immunodrugs [9]. Hydrogels, especially injectable hydrogels, have attracted attention as local and continuous drug delivery carriers for tumors. Compared with systemic drug delivery systems, injectable hydrogels have good injection ability and are minimally invasive. They can deliver a variety of anticancer drugs simultaneously in the tumor site, maintain a relatively high drug concentration, and reduce systemic toxic side effects. In this review, the applications of injectable hydrogels in tumor immunity in recent years were enumerated, and the development of the technology was prospected in four aspects: immunotherapy, chemoimmunotherapy, radioimmunotherapy, and photoimmunotherapy (Figure 1).

## 2. Hydrogels

### 2.1. Characteristics of Hydrogels

Hydrogel is a kind of biological material with unique porous structures that can swell in water or biological liquids. Based on its excellent properties, hydrogel has been utilized in drug release during chemoimmunotherapy [10], tissue engineering [11], wound dressing [12], and environmental engineering [13]. In immunotherapy, the hydrogel network allows the controlled and slow release of therapeutic drugs, nucleic acids, peptides, and therapeutic immune cell culture [10,14,15]. In situ, hydrogels formed by polymer solutions under mild conditions can deliver drugs locally, ensure the transport of sufficient drug concentration at tumor sites, and avoid serious systemic adverse reactions. Importantly, hydrogels have good biocompatibility, enzymatic and hydrolytic properties, and biocompatibility, induce low inflammatory response, and have promising in vivo applications [16]. Specifically, injectable hydrogels play a critical role in the delivery of tumor immunotherapy drugs. They can be directly injected into organisms through syringes or catheters. Generally, injectable hydrogels have shear-thinning characteristics or are in a liquid state before injection. After injection, they become suspended hydrogels through a physical/chemical crosslinking network [17]. Compared with invasive surgery, which still needs to be performed using an implantable system, the method of injection enables the drug to be delivered anywhere that the needle can reach, which is less invasive, and requires a shorter recovery time [18]. This largely avoids unnecessary tissue damage and complications related to inflammatory wound reaction, and the nursing process after injection is easier than after surgery. In addition, the injection of animals or human bodies usually requires less professional and technical knowledge, which is easier to achieve [19]. Furthermore, injectable materials can deform and flow to fit any available space before forming a permanent implant [20], which has significant advantages in clinical applications.

### 2.2. Classification of Hydrogels

Based on different standards, hydrogels can be divided into different types: (1) hydrogels can be divided into natural polymer hydrogels and synthetic polymer hydrogels, according to the different sources of raw materials. Natural polymer hydrogels are usually composed of natural polymers, such as proteins, polysaccharides, and nucleic acids. Natural polymer materials have unique advantages in biocompatibility, biodegradability, and environmental sensitivity, but they are not stable and are easily degraded [21]. Synthetic polymer hydrogels are usually synthesized using ring-opening polymerization. Synthetic hydrogels with good biocompatibility, such as polyacrylate and its derivatives [22], and polyethylene glycol and its copolymers [23], are usually used as materials in tissue engineering. The properties of synthetic hydrogels can be changed by adjusting the ratio and synthesis process, which makes them suitable for chemical modification and industrial production. However, compared with natural polymer hydrogels, synthetic hydrogels have poor biocompatibility, bioactivity and biodegradability [24]. Furthermore, hydrogels can be classified according to the mechanism underlying the formation of the 3D network structure. Chemically crosslinked hydrogels are mainly formed by introducing covalent bonds between polymer chains, including Schiff’s base reaction [25,26], Michael reaction [27], and the formation of disulfide bonds [28,29]. The process is irreversible once crosslinking occurs between molecules. Chemically crosslinked hydrogels have stable covalent crosslinking networks, so they have high mechanical strength and physical stability, a long degradation time, and an adjustable structure [30]. Meanwhile, physically crosslinked hydrogels are usually prepared using noncovalent interactions, such as hydrophobic interactions [31], hydrogen bonds [22], and ionic crosslinking [32,33]. Because the noncovalent bonds between molecules are easily destroyed, physically crosslinked hydrogels usually exhibit reversible sol–gel transformation behavior [34]. These hydrogels usually require only simple mixing without additional reagents during gelation, reducing the potential harmful effects on loaded biosurfactants/cells and surrounding tissues. Thermosensitive hydrogel is a typical example of a physically crosslinked hydrogel. It is very convenient to prepare in vitro drug-containing precursor solutions and inject into the body, and has been widely studied in various local tumor immunotherapies [15,35,36,37].

## 3. Tumor Immunotherapy

Immunotherapy has always been a promising strategy for cancer therapy and has achieved good results. However, only a small number of patients have achieved ideal therapeutic outcomes, and difficulties are still encountered when applying it to a large number of patients [9]. Limited immune response is still a major constraint that hinders the widespread application of immunotherapy [38]. Researchers have continuously explored new strategies to enhance the effect of immunotherapy. The injectable hydrogel system, which can control the release of therapeutic drugs in time and space, has attracted wide attention. At present, cancer immunotherapy includes three main strategies: adoptive cell therapy, immunomodulator therapy, and cancer vaccine therapy. This section will introduce the latest achievements on injectable hydrogels in immunotherapy based on the three strategies.

### 3.1. Immunomodulator

**Figure 2:**
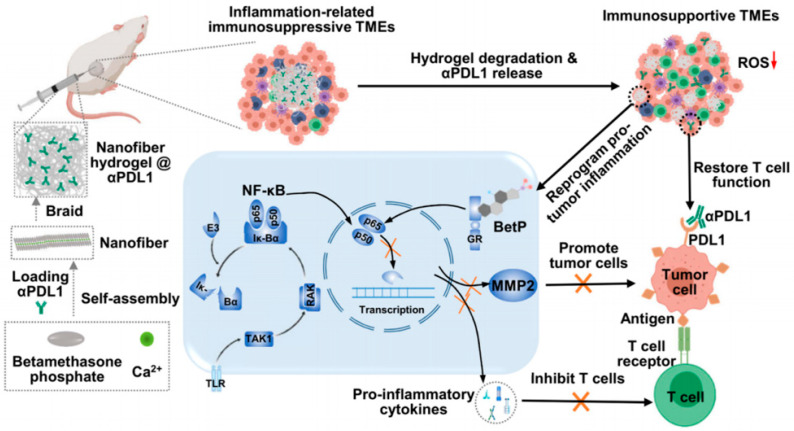
Schematic diagram showing the formation of nanofiber hydrogel through physical interaction between betamethasone phosphate and calcium ion. Such nanofiber hydrogels could transform the protumoral immunosuppressive tumor microenvironment (TME) to antitumoral TME by inhibiting the NF-κB signal pathway. Moreover, this hydrogel could sustainably release PDL-1 antibody to activate T cells, so as to synergistically boost the immune attack of tumor cells [42]. Copyright 2020, American Chemical Society.

Immunomodulators in immunotherapy include antibodies, cytokines, checkpoint blockade antibodies, etc. In recent years, immune checkpoint blockade (ICB) therapy has become increasingly prominent owing to its good therapeutic effect in cancer immunotherapy. Biological proteins activate immune checkpoints and negatively regulate T cell activation function to prevent excessive immune response in normal organisms [39]. However, tumor cells also use this feature to produce corresponding inhibition of T cell activation [40]. Immune checkpoint inhibitors block the inhibitory effect of immune cells by binding to the immune checkpoints. The most conventional way for ICB is to block the cytotoxic T lymphocyte antigen-4 (CTLA-4) and programmed death receptor-1/programmed death ligand-1 (PD-1/PDL-1) pathways, which can reactivate tumor-suppressed immune cells [39]. Nonetheless, a previous study found a significant increase in the risk of immune-related adverse events in patients treated with systemic checkpoint inhibitors [41]. Therefore, it is important to explore the reduction in ICB-antibody adverse reactions. Chen et al. [42] developed a nanofiber hydrogel loaded with a PDL-1 antibody (αPDL-1) to inhibit tumor growth (Figure 2). Calcium chloride aqueous solution was mixed with betamethasone phosphate disodium (BetP) solution to rapidly form betamethasone phosphate hydrogel (BetP-Gel), through simple physical crosslinking. Since betamethasone is as an anti-inflammatory steroid drug, BetP-gel can attenuate the nuclear factor kappa B (NF-κB) signaling pathway that is related to inflammation to reduce the expression levels of matrix metalloproteinase-2 (MMP2) and proinflammatory cytokines, and reactivate inflammation-related immunosuppressive TME. BetP-gel has a shear thinning, allowing it to be injected into the body to achieve locally controlled drug delivery. After loading αPDL-1 into the BetP-Gel, the αPDL-1 encapsulated BetP hydrogel (αPDL1@BetPGel) is formed, which can be gradually degraded and slowly released through the competitive interaction between phosphate and Ca^2+^ ions in hydrogels. The subcutaneous colorectal tumor model (CT26) showed significantly elevated levels of interferon γ (IFN-γ) in the tumors, indicating the T cell-mediated immune response was activated after using hydrogels, and significantly inhibited the growth of local tumors. In addition, a strong external absorption effect was observed in mice with distant tumors to treat metastatic cancer.

In another study, Cruz et al. [43] improved the immunotherapy effect of CTLA-4 by using 25% poloxamer 407 (P407) hydrogel to deliver CTLA-4 antibodies (αCTLA-4). P407 is a biocompatible polymer approved by the Food and Drug Administration (FDA). It is in a flow state below 18 °C and can be easily injected. Once injected into the body, P407 does not flow. In the CT26 colon cancer mouse model, the hydrogel containing αCTLA-4 effectively inhibited tumor growth without cytotoxic effects and improved the survival rate. After one week of treatment, the hydrogel in the body was clearly dissolved. In addition, in the MC-38 colon cancer model, the hydrogel loaded with CTLA-4 produced the same degree of tumor growth inhibition as in the CT26 model. Thus, further research using P407 hydrogel can be performed to deliver other immune checkpoint inhibitors or immunomodulators, rendering P407 to be a promising delivery system for optimizing tumor immunotherapy.

Herceptin (trastuzumab) is the first humanized monoclonal antibody drug against human epidermal growth factor receptor 2 (HER2), approved by the FDA in 1998 [35]. At present, Herceptin is mainly used in adenocarcinoma and metastatic gastric cancer with HER2 overexpression. However, trastuzumab has cardiotoxicity [44] and drug resistance [45]. Continuous local administration may be an effective strategy to improve therapeutic effects and minimize these systemic side effects. In a study by Lo et al. [46], they developed an injectable, chemically cross-linked hydrogel composed of γ-polyglutamic acid (γ-PGA-MA) and 4-arm poly(ethylene glycol) (4-ARM PEG-SH) to subcutaneously deliver trastuzumab. This new hydrogel has the ideal characteristics for loading a large amount of trastuzumab (>100 mg/mL) and being able to exist and release trastuzumab for several weeks in vivo to maintain a sufficient quantity of trastuzumab. The BT-474 breast cancer cell transplantation tumor model showed that the hydrogel controlled the release of trastuzumab and elicited the same antitumor effect as trastuzumab. In another similar study, Ding et al. [35] designed a thermosensitive injectable hydrogel system for the long-term release of Herceptin to successfully inhibit recurrence of HER2^+^ breast tumors. The hydrogel matrix poly (lactic-co-glycolic acid)- polyethylene glycol-poly(lactic-co-glycolic acid) (PLGA-PEG-PLGA) was prepared by a blending method, and the gel properties, in vitro and in vivo gel durability and drug release can be adjusted by simply changing the mixing ratio. Herceptin can be simply mixed with the polymer solution prepared using the optimal ratio in vitro, and this system can sustain Herceptin release for 80 days in vitro. Moreover, the tumor recurrence model established by using SK-BR-3 tumor-bearing mice to simulate breast-conserving surgery showed that the injection of Herceptin hydrogel near the tumor tissue improved the accumulation of antibodies in the tumor, which could inhibit tumor recurrence. This sustained release delivery system effectively prevented the cardiotoxicity caused by Herceptin.

Cytokines can recruit and activate dendritic cells (DCs), increase antigen presentation, and promote the expansion of T cells and natural killer cells [47]. Immunoreactions caused by the hydrogel delivery of cytokines into the body have also been reported. Langer et al. [48] developed a hydroxypropyl methylcellulose polymer nanoparticle (PNP) hydrogel loaded with DC cytokine C-C motif ligand 21 (CCL21). Injecting CCL21 into C57BL/6 mice subcutaneously can slowly and continuously release CCL21, recruit DCs to the injection site, initiate antigen presentation, and trigger antigen-specific T cells and antibody responses against selected targets. Compared with the non-CCL21-loaded PNP hydrogel, the CCL21-loaded PNP hydrogel preferentially recruits DCs into the injection site in vivo. However, this system only recruits DCs into the hydrogel, which is far from sufficient. Thus, further studies should be done using these findings; the addition of activators in the hydrogel and modifications to minimize the invasion in vivo should be performed. Meanwhile, Yano et al. [49] developed a hyaluronic acid-tyramine hydrogel mixed with interferon α (IFN-α) to treat renal cell carcinoma. IFN-α has immunoregulatory and direct antitumor properties, which are continuously released from hydrogels and prolong the action time to enhance the tumor inhibition effect to a certain extent. Sorafenib is a tyrosinase inhibitor that has been proven effective against renal cell carcinoma (RCC). In RCC-bearing mice injected with a hydrogel mixed with IFN-α combined with sorafenib oral administration, angiogenesis was inhibited, apoptotic cells increased, and tumor proliferation was inhibited. Although several positive results have been observed, the clinical efficacy of cytokine-based immunotherapy in the treatment of solid tumors in the past decade is still not very effective [50], and it still needs to be continuously explored.

In addition to the above immune checkpoint blockers, cytokines, and antibodies, some immune agonists/inhibitors and immune adjuvants have also been developed for the treatment of malignant tumors. Calcium/calmodulin-dependent protein kinase II (CAMKII) is carcinogenic in a variety of tumors and KN93 is its specific inhibitor [51]. Based on previous studies, Jin et al. [52] screened and synthesized a melittin-encapsulated hydrogel scaffold (MR52) with the best performance and loaded KN93 to form an MR52-KN93 (MRK) hydrogel. Due to the presence of melittin, the MR52 hydrogel has a direct killing effect on tumors while controlling drug release. Meanwhile, the MRK hydrogel killed tumor cells and improved the level of immunogenic cell death (ICD). Experiments show that MRK can reshape the TME, trigger a strong antitumor immune response, leading to the production of a large number of mature DCs in draining lymph nodes. Furthermore, MRK significantly enhanced the proportion of cytotoxic T cells and reduced the number of M2-like tumor-associated macrophages, which provided an effective treatment for melanoma and malignant ascites. The researchers also found that the expression of PD-L1 in macrophages increased after MRK treatment. The MRK hydrogel could further bind to the PD-1 antibody, and the cure rate of extensive peritoneal metastasis was approximately 30%.

Cyclic dinucleotides (CDNs) are considered stimulator of interferon gene (STING) agonists, which can activate the string downstream signaling pathway, induce the production of INFs, and initiate an anti-infection immune response [53]. CDNs have been used as a new type of vaccine adjuvant and immune drug. A representative example is the cyclic dinucleotide-loaded multidomain peptide hydrogel called “STINGel” developed by Hartgerink et al. [54] to enhance the efficacy of CDN immunotherapy. Multidomain polypeptides are supramolecular biological materials that can form nanofiber networks and solid hydrogels as delivery platforms. An MDP hydrogel with sequence K_2_(SL)_6_K_2_ was used to deliver CDN. STINGel released CDN eight times slower compared with the standard collagen hydrogel. In mouse oral cancer models, STINGel increased the survival rate by six times, but also improved immune memory and resisted a secondary attack of cancer cells in 100% of the surviving mice, compared with CDN alone or collagen hydrogel. Immune adjuvants are often used in cancer vaccine design and will be involved in the future development of other cancer vaccines.

### 3.2. Cellular Immunotherapy

**Figure 3:**
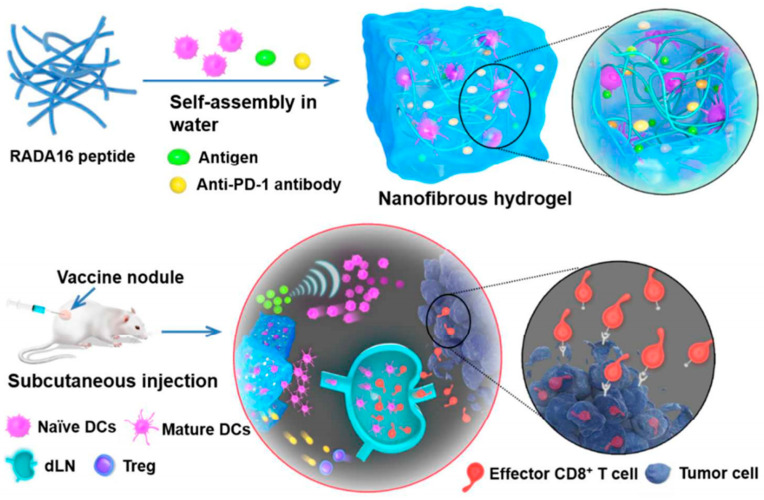
Formation and the mechanism underlying DC-based vaccine nodules engineered in the peptide nanofibrous hydrogel. In live mice, the hydrogel recruited and activated dendritic cells to migrate to lymph nodes, amplified antigen-specific spleen cells, and activated antitumor immune responses, which significantly delayed primary or recurrent tumor growth and prolonged survival [55]. Copyright 2018, American Chemical Society.

T cell-based targeted cellular immunotherapy has also become a hotspot in antitumor research recently. The patient’s own lymphocytes are usually reintroduced into the body after in vitro modification and amplification to promote an enhanced immune response. This strategy is often referred to as adoptive cell transfer therapy, represented by chimeric antigen receptor T cell (CAR-T) therapy. However, the direct systemic injection of expanded lymphocytes leads to insufficient localization at tumor sites. Local injection of hydrogels may solve this problem. In a study by Figdor et al. [15], an injectable biomimetic polyisocyanate (PIC) hydrogel was constructed for in vitro and in vivo delivery of immune cells. This study showed that this type of hydrogel could be used as a 3D culture system in vitro to support the survival and expansion of prestimulated T cells. Moreover, it would not affect the function of T cells even at a high cell density during the local transmission of preactivated T cells in vivo. Because of its thermal sensitivity, PIC hydrogel is a solution below 16 °C in vitro, so it can be locally injected into the body; the gel forms a stable scaffold so that the cells loaded in it are gradually released. In vivo, T cells in PIC hydrogel can reach remote organs at the same rate as T cells injected without scaffolds, while maintaining the proliferation and function of the T cells intact. In addition, PIC hydrogels have good biocompatibility, do not cause local or systemic inflammatory responses, and gradually degrade in vivo. In addition to T cells, PIC hydrogel can also support the viability of many other cells, which can be used as a valuable 3D cell culture system and cell delivery carrier. In addition, Wang [55] reported that a peptide nanofiber hydrogel loaded with DCs, anti-PD-1 antibodies and tumor antigens was prepared by simple physical mixing to improve the immunotherapy of malignant tumors (Figure 3). Hydrogels are assembled using RADA16 peptides consisting of periodic alternating hydrophilic and hydrophobic amino acids. After encapsulation by the hydrogel, DCs maintained their biological activity. Then, after subcutaneous injection in live mice, the vaccine nodules were able to recruit and activate large amounts of DCs to migrate to lymph nodes, amplify antigen-specific spleen cells, and activate antitumor immune responses. In EG7-OVA tumor-bearing mice, the treatment group that received hydrogel vaccine nodules had the highest efficacy in delaying the growth of primary or recurrent tumors and prolonging the survival rate of mice compared with other groups. Therefore, the developed combined immunotherapy mediated by vaccine nodules can effectively improve and penetrate tumor CD8+ T cells, inhibit the tumor immunosuppressive environment, and ultimately have good preventive and antitumor effects.

### 3.3. Cancer Vaccine

Cancer vaccines are often used in combination with other immune checkpoint inhibitors or immune adjuvants [56,57,58]. The combination of antigens and adjuvants has a synergistic effect on the immune response, which can cause an antigen-specific immune response with fewer side effects [59]. The slow-release properties of hydrogels extend the residence time of antigens and adjuvants at the injection site and stimulate stronger and longer-lasting immune responses. Wang et al. [57] reported an injectable hydrogel loaded with an RNA vaccine. Graphene oxide, with a particle size of approximately 200 nm, and low molecular weight polyethyleneimine formed a graphene oxide–low molecular weight polyethylenimine (GLP) hydrogel (GLP gel) through electrostatic interactions. The Toll-like receptor (TLR) 7/8 agonist Resiquimod (R848) and ovalbumin-encoded mRNA (mOVA) were enveloped in the hydrogel through π−π stacking and electrostatic interactions to form the GLP-R848 mOVA gel (GLP-RO gel). It is unstable at the interface between liquid and gel and gradually transforms into GLP-RO nanoparticles (GLP-RO NPs) when the GLP-RO gel is embedded in liquid solution. Therefore, after subcutaneous injection, GLP-RO NPs can be slowly released from the GLP-RO gel, and R848 and mOVA, which are translated into OVA antigen in antigen-presenting cells (APCs), are transported to the lymph nodes, effectively activating the immune system. In the B16-OVA melanoma model, GLP-RO Gel showed a good tumor inhibition effect and the released mOVA and R848 can produce tumor antigen antibodies in serum, thus preventing the formation of metastasis. In addition, mOVA can still be detected in C57BL/6 mice 30 days after in vivo injection; thus, the GLP-RO gel can effectively inhibit tumor growth, which requires only one treatment.

Lee et al. [36] developed a thermosensitive injectable hydrogel system that can be used to deliver DNA vaccines for cancer prevention. The hydrogel carrier (BSA-PCLA) is composed of a poly(ε-caprolactone-co-lactide)-b-poly(ethylene glycol)-b-poly(ε-caprolactone-co-lactide) triblock copolymer (PCLA) and bovine serum albumin (BSA) combined together using chemical crosslinking, producing a gel with a superior cell compatibility and safety than PCLA alone. BSA-PCLA implanted subcutaneously will form a nodule in situ and can be maintained for more than 10 days. The results showed that a single injection of DNA vaccine containing the BSA-PCLA mixed hydrogel was sufficient to cause humoral immunity. Hydrogels convene a large number of immune cells through inflammatory reactions, and the 3D network structure provides storage space for immune cells. This allows for a continuous and slow delivery of the pDNA vaccine to promote the in situ programming of DCs. Furthermore, the authors mixed the polybody containing Aβ DNA vaccine (pTarget-Ig-Aβ-Fc) in this thermosensitive hydrogel and implanted them into the back of mice for the test. The results showed that the Aβ DNA vaccine delivered by this strategy was very effective in reducing Alzheimer’s disease. Thus, the BSA-PCLA is a promising vaccine delivery system. The use of human serum albumin (HSA)-based hydrogels as vaccine carriers may be safer. The potential of HSA-PCLA as a vaccine remains to be explored.

Granulocyte-macrophage colony-stimulating factor (GM-CSF) is an immune adjuvant that can induce the proliferation and differentiation of APCs, such as DCs or macrophages, and is often used for cancer immunotherapy. Qian et al. [37] designed a hydrogel vaccine system to deliver immunomodulators and antigens during cancer vaccination. The thermosensitive poly(D, L-lactide)-poly(ethylene glycol)-poly(D, L-lactide) (PDLLA-PEG-PDLLA, PLEL) triblock copolymer self-assembled into micelles in water and coated with tumor cell lysates, GM-CSF, and TLR9 agonist cytosine-phosphate-guanine oligonucleotide (CpG-ODN) to form a hydrogel vaccine system, which forms a gel at 37 °C. This hydrogel slowly and continuously releases antigens and adjuvants in the tumor area, promoting the maturation and activation of bone-marrow-derived DCs. On the 14th day after immunization, the expression of CD86 and a major histocompatibility complex I (MHC-I) on the surface of DCs and the level of serum tumor necrosis factor (TNF) were significantly increased, compared with the blank group. In addition, in the B16F10 and CT26 tumor-bearing mice models, tumor growth was inhibited to a certain extent after injection of the hydrogel. Therefore, the hydrogel vaccine has significant preventive and therapeutic potential in eliminating various tumors and has high clinical application value. In the future, adjusting the types of antigens and immunomodulators in this system is expected to achieve a broader treatment and stronger tumor immune response. In another similar study, Wang et al. [60] used mPEG-block-poly (L-alanine) copolymer as a platform to load tumor vaccines composed of tumor cell lysates, GM-CSF, and double immune checkpoint inhibitors to prepare peptide hydrogel delivery systems. GM-CSF was used as an adjuvant to induce a T cell-mediated immune response. In particular, the addition of immune checkpoint inhibitors anti-CTLA-4 antibody and anti-PD-1 antibody further enhanced the antitumor effect. In the 4T1 tumor and B16 melanoma models, not only the production of IgG and the secretion of cytokines, such as IFN-γ, interleukin-4 (IL-4), and TNF-α, were upregulated; the effector T cell CD8^+^ was activated, and the number of Tregs was reduced. The peptide hydrogel combined with immunotherapy has a good therapeutic effect on B16F10 melanoma and 4T1 breast cancer. It has a certain specificity for B16 and provides a promising tumor treatment model. Moreover, tumor cell lysates prepared from B16 cells were used as antigens. If other types of antigens are used, they might have a wider application range. This result is based on a previous study by Wang et al. [56] who used self-assembled polyethylene glycol peptide hydrogel as a tumor vaccine delivery system for the first time to regulate host DCs to achieve antitumor effects. The hydrogel matrix mPEG-block-poly(L-alanine) (PEV) copolymer was synthesized using ring-opening polymerization of L-adenine and mPEG-NH2. Afterwards, this polypeptide hydrogel tumor vaccine delivery system can be prepared by simply mixing tumor cell lysates (TCL) prepared by B16 tumor cells and TLR3 agonist polyinosinic: polycytidylic acid (poly (I:C)) with PEV copolymer in aqueous solution, which can continuously release antigens or poly (I:C) for more than one week, prolong the antigen retention time at the injection site, and increase the percentage of antigen drainage to lymph nodes. It can regulate DCs in vitro and in vivo. The experimental results showed that in the B16 melanoma mouse model, the polypeptide hydrogel vaccine formulation containing TCL and poly (I:C) effectively induced the migration of activated DCs to drainage lymph nodes (dLNs), and inhibited tumor growth by stimulating the strong cytotoxic T lymphocyte response in dLNs and enhancing the expansion of tumor-infiltrating lymphocytes. Our results show that the dual transfer of peptide hydrogel-based antigens and TLR3 agonists to regulate host DCs in vivo is an effective method for direct cellular immunity against cancer. A simple preparation process, convenient injection, and good biocompatibility have great potential for clinical transformation.

## 4. Injectable Hydrogel for Local Chemo-Immunotherapy Combination

Chemotherapy, as one of the traditional methods of cancer treatment, can treat some diseases, but serious side effects increase the psychological and physiological burden in patients [61]. Studies have shown that chemotherapy can kill tumor cells through cytotoxicity [62] and activate the immune system in various ways. For example, some chemotherapeutic drugs produce damage-associated molecular patterns (DAMPs) through killing tumor cells, then DAMPs can activate T cell-mediated adaptive immune response like “tumor vaccine”. This process is called immunogenic cell death [63]. Appropriate immunotherapy combined with chemotherapy has a better antitumor efficacy than a single therapy. This section will mainly introduce the combination of Adriamycin (DOX) and camptothecin (CPT) with immunotherapy drugs.

Li et al. [64] developed a thermoresponsive injectable immunomodulatory hydrogel that blocks the Arginase 1 (ARG1) pathway. A diblock copolymer of PLN-PEG was formed through self-assembly based on the inhibitor L-n-alanine of the ARG1 pathway. The formed polypeptide block-polyethylene glycol (PLN-PEG) molecular solution can be rapidly transformed into hydrogel after being injected into the tumor site, which detects the high drug loading and gradual release of L-valine under the action of peptide degradation enzymes. This effectively solved the problem of rapid release and high dosage caused by the high-water solubility of L-valine. The authors introduced ICD-induced DOX into a PLN-PEG gel to form a PLN-PEG@DOX hydrogel. In a B16F10 mouse model, the combination of DOX-induced ICD and the reversal of the ARG1 immunosuppressive environment promotes the maturation of DCs, induces the internal filtration and accumulation of a large number of lymphocytes in tumors, effectively inhibits the growth of primary tumors, and generates systemic immune responses. This immunoregulatory hydrogel system provides a new approach for improving the TME in immunosuppression and enhancing the effect of immunotherapy. In another study, Yang et al. [65] developed a supramolecular hydrogel that encapsulated DOX and ^D^PPA-1, a D-peptide antagonist with high affinity to PD-L1, for combined chemotherapy and immunotherapy. Drug-containing hydrogels injected into the tumor site can gradually release DOX and ^D^PPA-1, which play a synergistic role in enhancing the infiltration of cytotoxic CD8+ T cells in tumors and inducing the secretion of TNF-α and IFN-γ at the highest level. In the CT26 colorectal tumor subcutaneous model, significant tumor inhibition was observed.

Lv et al. [66] built a dual fluorescence visualization delivery system. A simple mixture of PEG and α-cyclodextrin was used to prepare the hydrogel, which was wrapped with DOX and immune adjuvant cytosine–phosphate–guanine (CpG) self-crosslinking nanoparticles (CpG NPs) for immunotherapy of tumor chemotherapy. The sustained release of the hydrogel maintained the effects of DOX chemotherapy and CpG NP-enhanced immunotherapy. The two compounds showed synergistic effects, signaling the TME to positively regulate tumor inhibition. Dual fluorescence imaging allows the release, distribution, and metabolism of DOX and CpG nanoparticles to be visually monitored. In other studies, DOX and two cytokines were co-loaded into hydrogels for chemotherapy combined with immunotherapy. In a typical example, Chen et al. [67] developed a biodegradable polypeptide thermosensitive hydrogel for melanoma treatment. Poly (g-ethyl-L-glutamate)-poly (ethylene glycol)-poly (g-ethyl-L-glutamate) (PELG-PEG-PELG) was synthesized through the ring-opening polymerization of g-ethyl-L-glutamate-N-carboxylic acid hydride initiated by NH_2_-PEG-NH_2_. The hydrogel system was loaded with DOX, IL-2, and IFN-γ cytokines, which were released rapidly and massively in vivo at first, and then released slowly for 26 days. In a nude mouse model, this co-delivery strategy had a good therapeutic effect on melanoma and no obvious side effects.

**Figure 4:**
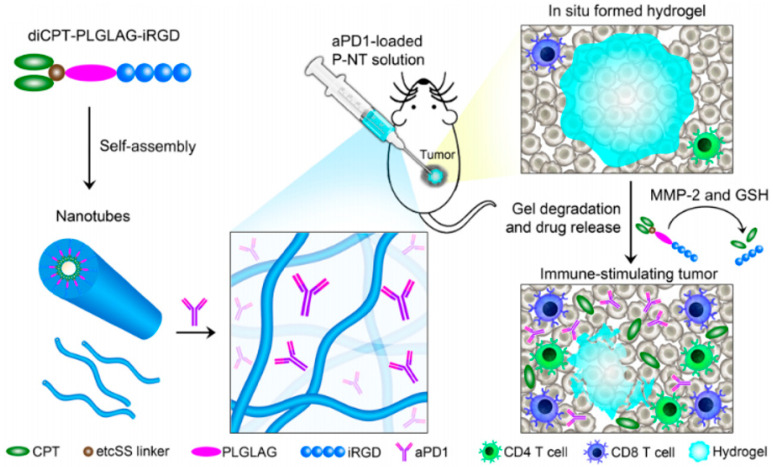
Schematic illustration of local codelivery of CPT and aPD1 using an in situ formed supramolecular hydrogel to attain bioresponsive drug release and tumor microenvironment regulation. This in situ hydrogel can slowly deliver antibodies, and glutathione, which is widely present in tumors, can break the disulfide bonds of etcSS, freeing CPT. Thus inducing a powerful T cell-mediated tumor immune response [69]. Copyright 2020, the American Association for the Advancement of Science.

Camptothecin (CPT) is a two-molecule hydrophobic natural cytotoxic antitumor drug that inhibits DNA topoisomerase I-induced DNA damage and induces cell death [68]. However, the toxicity of CPT to normal tissues limits its clinical application. Various methods have been explored to reduce their side effects. Cui et al. [69] reported a carrier-free prodrug hydrogel delivery system (Figure 4). CPT was linked with a hydrophilic tumor-penetrating peptide iRGD (a tumor-penetrating peptide that can bind to neuropilin-1 (NRP-1) and trigger tumor tissue penetration) through an MMP2 response connector (plglg peptide) and a reducible disulfide-ethyl carbonate (etcSS) connector to form supramolecular nanotubes (P-NTs) in water. The mixture of αPD1 and P-NTs in the tumor site can form an in situ hydrogel for the slow transmission of antibodies, and the glutathione widely existing in the tumor can destroy the disulfide bond of etcSS and release CPT. The P-NT-αPD1 hydrogel induced a strong T cell-mediated tumor immune response. In the GL-261 brain cancer and CT-26 colon cancer models, all tumors in the treated mice showed 100% regression in systemic antitumor immunity and memory immune response in GL-261 tumors. In another study by the same group, a self-assembled supramolecular hydrogel was developed to promote the delivery of a STING agonist (c-di-AMP (CDA))and CPT in tumors for chemotherapy-immunotherapy of malignant tumors [70]. A peptide-drug conjugate (diCPT-iRGD) was synthesized by coupling iRGD with CPT at a ratio of 1:2 in aqueous solution. This amphiphilic conjugate can form nanotubes (NTs) in aqueous solution. Then CDA binds to the surface of nanotubes through electrostatic interaction to form CDA-NTs. When the CDA-NT solution is injected into the tumor site, the hydrogel automatically forms as a drug repository, which triggers the activation of the immune response. The survival rates of GL-261, 4T1, and CT26 tumor mice were significantly increased after using this hydrogel. In addition, the supramolecular hydrogel can also prevent tumor recurrence and metastasis. However, this supramolecular hydrogel synthesis process is relatively complex, which limits its application to some extent.

The combination of other chemotherapeutic drugs and immunotherapy has also been studied. Gu et al. [71] developed a reactive oxygen species (ROS)-responsive hydrogel for the local delivery of chemotherapy and immunotherapy with gemcitabine (GEM) and αPDL-1. Hydrogel scaffolds are formed by the cross-linking of polyvinyl alcohol (PVA) with N^1^-(4-boronobenzyl)-N^3^-(4-boronophenyl)-N^1^,N^1^,N^3^,N^3^-tetramethylpropane-1,3-diamini-um (TSPBA), an ROS-unstable linker that can be used for drug storage and reduce ROS in TME to improve the therapeutic effect. The hydrogen peroxide level in the TME is high, and the in situ formed αPDL1-GEM@Gel gradually degrades and releases GEM and αPDL-1. GEM induces PD-L1 expression, which enhances the effect of αPDL-1. In mouse models of B16F10 melanoma and 4T1 breast tumors, they all showed systemic tumor inhibition, and the formation of memory T cells prevented tumor recurrence. Gu et al. [72] designed a bioresponsive hydrogel = to acidic pH and ROS in the TME to co-deliver zebularine (Zeb) and αPDL-1. The αPDL-1-loaded pH-sensitive calcium carbonate NPs and Zeb were encapsulated in ROS-responsive hydrogels. Then the formed hydrogel could simultaneously release Zeb and αPDL-1 under acidic pH and ROS. The released Zeb regulates the expression of tumor-associated antigens (TAAs), reverses the immunosuppressive TME, upregulates the expression of PDL-1, promotes the release of αPDL-1, and induces a strong antitumor immune response. The experiment showed that the combined treatment was helpful in inhibiting the tumor growth of B16F10 melanoma mice and prolonged their survival time. The long-term toxicity of these reactive hydrogels should be assessed thoroughly. In addition, the dose and frequency of different therapeutic drugs need to be further optimized. Several reports on the combination of chemotherapy drugs and immunotherapy in hydrogels have achieved good antitumor effects. However, these hydrogels are mostly synthesized by chemical crosslinking, and their long-term toxicity in vivo remains to be tested.

## 5. Injectable Hydrogel for Local Radio-Immunotherapy Combination

Radiotherapy using radioisotopes or external ionizing radiation to destroy tumors has been widely used in clinical cancer treatment. It can induce immunogenicity in cancer cells. Radiotherapy combined with immunotherapy not only improves the local efficacy of radiotherapy, but also enhances the distant antitumor effect [33]. For example, radiotherapy combined with ICBs has been proven to be effective in the treatment of some cancers, such as breast cancer and melanoma [73,74], while systemic administration of radioisotopes can lead to toxic effects in the normal tissues and organs of patients. Hydrogel is a local drug delivery platform for a radiotherapy and immunotherapy combined therapy, which greatly reduces side effects.

Liu et al. [33] designed an in situ injection hydrogel based on sodium alginate (ALG) combined with radiotherapy and immunotherapy to effectively combat malignant tumors. In this study, the authors selected ALG as a carrier, which is in liquid state in vitro, because it can be injected into the tumor and cross-linked under the physiological concentration of Ca^2+^ to form an in situ hydrogel without leaking in other normal tissues. The ^131^I-labeled catalase (^131^I-Cat) was mixed in ALG and injected into the tumor site, resulting in the decomposition of hydrogen peroxide in the tumor, which improved the oxygenation status of the tumor. A 100% local tumor elimination was achieved in 4T1 mouse breast cancer, human prostate cancer patient-derived xenograft mice, and larger VX2 liver cancer rabbit models. Subsequently, the authors added CpG as an immune adjuvant on the basis of this system, which significantly inhibited the growth of distal tumors in the colorectal CT26 tumor model of mice and prevented tumor recurrence to a certain extent. This indicated that ^131^I-Cat/CpG/ALG treatment triggered a systemic antitumor immune response. Finally, the combination of ^131^I-Cat/CpG/ALG and αCTLA-4 also showed a significant synergistic effect. In summary, this strategy is not only suitable for the elimination of large solid tumors, but also for eliminating metastatic tumors and preventing tumor recurrence; thus, it is expected to be applied in a wider range of tumors. In another study, they reported a radioimmunotherapy combined with antitumor therapy for hydroxy propyl cellulose (HPC) hydrogel-loaded IFN-α2b (Gel-IFN) combined with intraperitoneal T cell injection and low-dose X-ray irradiation (LDI) [75]. They performed a low-dose X-ray irradiation on tumor tissue and then injected T cells into the tumor site. Finally, IFN-α2b released slowly by the hydrogel was used as an enhancer to fully activate T cells and cause tumor killing. LDI promoted the aggregation of T cells in tumors, while IFNα-2b induced the activation of T lymphocytes and increased IFN-γ secretion. The results showed that the combination of Gel-IFN, T cells, and LDI resulted in the smallest tumor volume in human gastric cancer MKN-45 subcutaneous transplantation mouse model, which had excellent antitumor effects.

## 6. Injectable Hydrogel for Local Photo-Immunotherapy Combination

Phototherapy using light (usually near infrared (NIR) light) to stimulate photosensitizer/photothermal agent-induced cell death has been developed in recent years. Its unique characteristics of light triggering and noninvasiveness have attracted attention; thus, it has been used for cancer treatment [76,77]. Phototherapy includes photothermal therapy (PTT) and photodynamic therapy (PDT) according to the different mechanisms. Hydrogels for phototherapy have also been developed [78,79]. Single therapy often has limitations, and photoimmunotherapy (PIT) combined with immunotherapy has been proposed. Unlike other traditional therapies, PIT not only weakens the host’s antitumor immune response, but also activates specific antitumor immune responses. Phototherapy can cause the ICD of tumor cells and then quickly release tumor-specific antigens as an in situ autologous vaccine. The released DAMPs can stimulate the immune response, thereby attracting DCs to migrate to the tumor site and inducing them to present tumor-specific antigens, activating tumor-specific T cells to proliferate and mediate tumor cell death. At the same time, immunotherapy increases the number of cytotoxic CD8+ T cells and affects memory T cells in tumor infiltration by adjuvant-assisted recruitment of more DCs in the TME, or by blocking immune checkpoints to reduce immune regulation inhibition [80,81]. However, excessive activation of the immune system caused by systemic administration may lead to adverse reactions. Local administration is the ideal method for postoperative treatment [82,83]. Locally injectable hydrogels are an ideal choice for local drug release. Several injectable hydrogels that combine phototherapy and immunotherapy for cancer have been developed.

### 6.1. Combination of PTT and Immunotherapy

PTT is a therapeutic strategy for tumor ablation using NIR light to stimulate photothermal agents, rapidly converting light energy into heat energy, and improving local tumor temperature [84]. The single use of photothermal therapy for treatment cannot completely treat tumors generally and is likely to cause uneven heat distribution in tumor tissues. The combination of photothermal therapy and immunotherapy can greatly improve the efficacy of tumor vaccines and improve the efficacy of immunotherapy. Immunoadjuvants are often used in combination with photothermal agents because they can co-activate immune responses with TAAs produced by tumor ablation to improve the efficacy of immunotherapy. In a study of Lv et al. [66], a dual-channel self-fluorescent CpG self-crosslinked nanoparticle loaded new indocyanine green (IR820) conjugate hydrogel was synthesized. CpG and IR820 exhibited fluorescence characteristics. Dual fluorescence is helpful for observing the release, distribution, and interaction of these two adjuvants in vivo, which provides a possibility for the accurate treatment of tumors. The IR820 hydrogel can effectively conduct hyperthermia to eliminate primary tumors, and the generated tumor antigen is used for adjuvant immunotherapy in the meantime. CpG self-crosslinking nanoparticles enhanced the immune response of adjuvants to melanoma by increasing drug loading and extending CpG release time. By combining CpG self-crosslinking nanoparticles with the IR820 hydrogel, the systemic therapeutic effect is more effective than photothermal therapy or immunotherapy alone. It has been reported that this treatment can increase the proportion of DCs, B cells, and CD8+ T cells in the TME and reduce the number of Treg cells and Myeloid-derived suppressor cells (MDSCs). This CpG NPs/IR820 hydrogel-mediated combination therapy completely eradicates tumor residues and is more effective than PTT or immunotherapy alone. The therapeutic effects of a single adjuvant are often limited. In another study, Wei and Qian [85] developed a thermoresponsive injectable hydrogel containing multifunctional self-assembled NPs, which can be used for local PTT-immunotherapy by regulating near-infrared light-controlled release drugs. In this platform, indocyanine green (ICG) composed of physically bound self-assembled NPs (called RIC NPs) was used as a photothermal agent, and R848 and CpG ODNs were transferred to thermosensitive PDLLA-PEG-PDLLA (PLEL) hydrogels. The formed RIC NPs@PLEL hydrogel was injected into the tumor resection cavity. Under the irradiation of 808 nm near-infrared laser, ICG exerted PTT to perform photothermal ablation of tumor residues to generate TAAs. In addition, the local heat generated resulted in the release of R848 and CpG ODNs from the thermoresponsive hydrogel, which was synergistic with TAAs to generate strong antitumor immunity. In this way, this treatment promotes DC maturation, T cell propagation, and cytokine secretion. In 4T1 tumor-bearing mice, this dual adjuvant-loaded hydrogel-mediated photothermal immunotherapy effectively inhibited lung metastasis and prevented local tumor recurrence. The synthesis of thermosensitive PLEL hydrogels is a complex process, and its long-term biocompatibility needs to be carefully evaluated. In a recent study by Oh et al. [86] designed a biodegradable DNA hydrogel (Mel/G/DH), wherein bis-(3′-5′)-cyclic dimeric guanosine monophosphate (G/DH) was loaded onto a DNA hydrogel (DH) containing CpG sequence produced by rotating circular amplification (RCA), and melanin was coated as a light-responsive component. In vitro studies showed that under 808 nm irradiation, Mel/G/DH significantly induced the calreticulin exposure on the surface of CT26 cells and activated the maturation of DCs. In vivo, the effect of this hydrogel system on DNAse was degraded, and the synergistic effect of the TLR9 agonist CpG and STING agonist c-di-GMP co-transmission was observed. In the CT26 mouse model, the photothermal effect generated by NIR radiation completely ablated the primary tumor, promoted the release of tumor antigen in the mouse model, induced the maturation of DCs in lymph nodes, regulated the systemic immune response, and prevented the recurrence of distal tumors. In addition, Mel/G/DH light immunotherapy can also reshape the immune microenvironment of distant tumors, increase cytotoxic T cells, and reduce Treg cells, with a 100% survival rate in mice.

**Figure 5:**
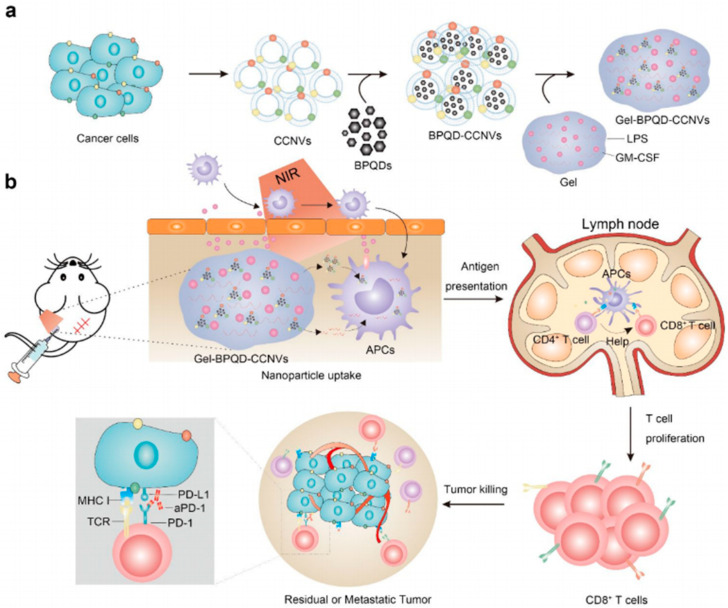
(**a**) Preparation of Gel-BPQD-CCNVs. (**b**) Mice after surgery were injected subcutaneously with Gel-BPQD-CCNVs and irradiated with NIR to recruit dendritic cells. The nanoparticles were internalized by DCs and neoantigens presented to T cells to enable T cell activation. The residual and metastatic cancer cells were killed by activated T cells. Anti-PD-1 antibody was used to prevent the tumor immune evasion caused by the tumor PD-L1 ligand [87]. Copyright 2019, American Chemical Society.

In addition, the immune adjuvant GM-CSF was also used to prepare PTT combined with an immune therapy hydrogel. For instance, Mei et al. [87] developed a personalized photothermal vaccine that integrates black phosphorus quantum dot nanovesicles (BPQD-CCNVs), GM-CSF, and lipopolysaccharide (LPS) coated with 4T1-luc or B16F10-luc cancer cell membranes into a thermosensitive hydrogel to form a subcutaneous tumor vaccine (Figure 5). The continuous release of GM-CSF from subcutaneously injected gel BPQD-CCNVs effectively recruits dendritic cells to capture tumor antigens. NIR radiation and LPS stimulate the expansion and activation of DCs and then reach the lymph nodes to present antigens to CD8+ T cells. Compared with the traditional single antigen vaccine restricted by individual epitope identification, vaccines based on cancer cell membranes can provide abundant autologous tumor antigens. Moreover, continuous release of GM-CSF from subcutaneously injected gel BPQD-CCNVs effectively recruits DCs to capture tumor antigens.

In addition to immune adjuvants, immune checkpoint inhibitors have also been loaded into hydrogels for photoimmunotherapy. Sun et al. [88] prepared an injectable lipid hydrogel (LG) using soybean phosphatidylcholine and glycerol dioleic acid, which was loaded with IR820 dye and αPDL-1 and had photothermal sensitivity and reversible formation ability. After local injection to the tumor site, the lipid mixture was hydrated to form a gel library (αPDL-1/I@LG), and a reversible gel–sol transition occurred at approximately 39 °C. Under an 808 nm laser irradiation, IR820-mediated PTT generated mild heat locally, leading to LG phase transition; thus, the release of αPDL-1 can be controlled by adjusting irradiation. In addition, a mild PTT effect can trigger a systemic immune response, promote T cell infiltration into tumors, and upregulate the expression of αPDL-1 in the TME. Therefore, this strategy of treatment effectively inhibited the growth of both primary and distant tumors, improved the rate of animal survival, and prevented lung metastasis in 4T1 and B16F10 tumor models. Although it has been reported that LG degrades after subcutaneous injection in live mice, its long-term biological safety needs to be further evaluated in vivo.

Mixed hydrogels containing antitumor drugs, photothermal agents, and immunotherapy have also been reported. By coencapsulating near-infrared absorption silver sulfide (Ag_2_S) QD, DOX, and bestatin (an immune adjuvant) in polypeptide nanogels (PC10ARGD), Liu et al. [89] prepared an in situ injected vaccine for breast cancer treatment. Under irradiation with an 808 nm laser, the PTT effect mediated by Ag_2_S QD triggered the continuous release of DOX and initiated in situ immunity together with PTT. The bestatin released from hydrogels as an immune adjuvant improved antitumor immunity by improving T lymphocyte function. This treatment mediated by multifunctional hydrogel increased the infiltration of CD8+ T lymphocytes in the tumor site, and the high levels of cytokines IL-12p70 and IFN-γ in the serum, which can significantly inhibit the growth of primary tumors and the occurrence of distal pulmonary metastasis nodules. However, the toxicity of Ag_2_S QDs should be addressed to facilitate further application. In addition to these hydrogels, a physiologically triggered injectable red blood cell-based cancer photoimmunotherapy hydrogel has been developed. Wang et al. [90] found that homologous red blood cells can spontaneously form hydrogel-like components in situ after subcutaneous injection in mice. This is due to the red blood cells being triggered by physiological signals such as platelets and thrombin. These components do not produce unknown side products during metabolism or degradation in vivo. At the same time, the photothermal effect of an in situ formed red blood cell hydrogel irradiated by an 808 nm infrared laser led to the release of the TLR7 agonist imiquimod (R837) from red blood cells. R837 was loaded into red blood cell gel (R837@RBCs) by hydrophobic interaction and injected into CT26 colon cancer xenografts in mice. The photothermal effect of this gel can effectively burn tumors to release tumor-associated antigens, promote the release of R837 to nearby lymph nodes, and induce the activation of a large number of APCs. The combined treatment of primary tumors can cause persistent systemic immune responses to restrain the metastatic tumors’ growth. The mice activated by laser with R837@RBCs could resist tumor recurrence, and no tumor was found after survival for at least 250 days.

### 6.2. Combination of PDT and Immunotherapy

PDT generates ROS through the absorption of light at a specific wavelength by a photosensitizer, resulting in oxidative damage to tumor cells. PDT has been proven to be an effective treatment for many cancers [91]. Similar to PTT, PDT not only causes tumor cell death through ROS, but it also initiates an immune response to tumor cells [92].The combination of PDT and immunotherapy has been widely studied, and photoimmune injectable hydrogels containing photosensitizers and immune adjuvants have been reported.

Imiquimod (R837) is a TLR7 agonist. Liu et al. [93] reported a photo-triggered in situ formed hydrogel system in one of their studies. This system contains Ce6-modified catalase, R837-loaded poly(lactic-co-glycolic acid) nanoparticles (RPNPs) as an immune adjuvant, and PEG diacrylate (PEGDA) as a polymer matrix. The mixed precursor solution was injected locally into the tumor. Under a 660 nm laser irradiation, ROS generated by the Ce6 photoinitiator could induce PEGDA to form a hydrogel in situ. Catalase encapsulated in the in situ hybrid hydrogel triggers endogenous hydrogen peroxide decomposition to produce oxygen, which continuously alleviates tumor hypoxia. This process improves the efficacy of PDT and reverses the immunosuppressive tumor microenvironment at the same time, which is conducive to antitumor immunity. PDT was generated using multiple rounds of irradiation with a 660 nm laser, and ICD was induced by releasing TAAs. TAAs and RPNPs have been used as immune adjuvants to stimulate strong antitumor immunity. This treatment strategy has a significant effect on inhibiting tumor growth and prolonging the survival time of the 4T1 tumor-bearing BALB/c mouse model. The authors further combined hydrogel-based multi-round PDT strategy with αCTLA-4-mediated immunotherapy, which not only completely ablated primary and distant tumors, but obtained sufficient long-term immune memory to reduce the risk of tumor recurrence. In a future study, we will attempt to load immune checkpoint antibodies into the hydrogel.

Sun et al. [94] developed a hydrogel for chargeable photodynamic tumor immunotherapy to achieve a significant photodynamic-immune synergistic effect. Light-emitting material (PLM) with bright red luminescence properties and immune adjuvant R837 were introduced into alginate-Ca^2+^ hydrogel to prepare an immune hydrogel (PLM-R837-ALG hydrogel, PRA). This hydrogel can be injected into the tumor site. This gel does not leak and spread to the surrounding tissue. PLMs exhibit strong persistent luminescence and rechargeability that can effectively activate the photosensitizer Ce6, resulting in continuous PDT. The release of R837 can effectively amplify the immunogenicity of antigens that are generated during PDT, thus leading to a strong immune response and the killing of tumor cells. In in vitro experiments, the PRA hydrogel effectively activated DCs and induced strong immune responses. In 4T1 tumor mice, tumor growth was remarkably inhibited after photodynamic immunotherapy, and LED irradiation did not cause damage to other tissues.

## 7. Conclusions

Currently, the field of cancer immunotherapy is developing at a fast rate. Although immunotherapy and combination therapy provide therapeutic effects, several side effects or toxic effects are still observed. An injectable hydrogel as a local drug delivery carrier may be a good solution to enhance local drug concentration in tumor tissue and reduce drug distribution throughout the body. This study reviews the latest progress in injectable hydrogels as drug carriers in tumor immunotherapy and combined therapy. After the immunotherapeutic drugs were loaded into several kinds of hydrogels, they had a significant therapeutic effect on tumors. With the in-depth study of hydrogels and the continuous improvement of immunotherapy and other combined therapies, hydrogels can also load chemotherapy drugs, radioisotopes, and photoresponsive agents to mediate chemotherapy, radiotherapy, and phototherapy combined with tumor immunotherapy. Ideal results were also observed. Nevertheless, different treatment strategies have advantages and disadvantages. Table 1 summarizes the characteristics of the different treatment regimens. First, most hydrogels are composed of synthetic polymers. Although a few polymers have been certified by the FDA, the long-term safety of hydrogels composed of polymers in vivo still needs attention. Second, the tissue penetration ability of NIR light is limited, and photoimmunotherapy can only be used for superficial tumors. Third, the dosage of drugs and the toxicity of radioactive substances in radiotherapy combined with immunotherapy need to be determined and considered. Fourth, based on the use of injectable hydrogels, most of these gels are still used for the treatment of superficial tumors (such as melanoma and breast tumors) and are difficult to use in special organs (such as the lungs), which makes it impossible for use as a broad-spectrum antitumor treatment. Finally, the current studies are based on in vivo mice experiments, and there is a gap with the actual human tumor pathological model. Although there is still a long way to go for the clinical transformation of injectable hydrogel-mediated tumor local immunotherapy, the cooperation between scientists in different disciplines can explore more reasonable joint programs and elucidate the potential of hydrogels in cancer treatment.

## Figures and Tables

**Table 1:** Characteristics of the different cancer treatment regimens.

Therapeutic Strategy	Advantage	Disadvantage	Phase	Reference
Immunotherapy	Simple hydrogel composition and synthesis process	Limited therapeutic effect	Preclinical step	[35,42,43,46,48] [15,49,51,53,54] [36,37,56,59,87]
Chemoimmunotherapy	High immune response, better treatment effect	Hydrogels are usually complex in composition and their long-term biosafety in vivo needs to be confirmed	Preclinical step	[63,64,65,66,68] [69,70,71]
Radioimmunotherapy	High immune response, better antitumor effect at the distal end	Safety of radioactive elements	Preclinical step	[33,75]
Photoimmunotherapy	High immune response, good therapeutic effect, and great time and space control	The limited penetration of near infrared light to tissues and the toxicity of photosensitizers	Preclinical step	[65,83,84,85,86] [89,93,94]

## References

[1] Siegel R.L., Miller K.D., Jemal A. (2020). Cancer statistics, 2020. CA Cancer J. Clin..

[2] Wang H., Mooney D.J. (2018). Biomaterial-assisted targeted modulation of immune cells in cancer treatment. Nat. Mater..

[3] De Ruysscher D., Niedermann G., Burnet N.G., Siva S., Lee A.W.M., Hegi-Johnson F. (2019). Radiotherapy toxicity. Nat. Rev. Dis. Prim..

[4] Song W., Kuang J., Li C.-X., Zhang M., Zheng D., Zeng X., Liu C., Zhang X.-Z. (2018). Enhanced Immunotherapy Based on Photodynamic Therapy for Both Primary and Lung Metastasis Tumor Eradication. ACS Nano.

[5] Li S., Zhang Z., Lai W.-F., Cui L., Zhu X. (2020). How to overcome the side effects of tumor immunotherapy. Biomed. Pharmacother..

[6] Duan Q., Zhang H., Zheng J., Zhang L. (2020). Turning Cold into Hot: Firing up the Tumor Microenvironment. Trends Cancer.

[7] Song Q., Zhang G., Wang B., Cao G., Li D., Wang Y., Zhang Y., Geng J., Li H., Li Y. (2021). Reinforcing the Combinational Immuno-Oncotherapy of Switching “Cold” Tumor to “Hot” by Responsive Penetrating Nanogels. ACS Appl. Mater. Interfaces.

[8] Yan S., Luo Z., Li Z., Wang Y., Tao J., Gong C., Liu X. (2020). Improving Cancer Immunotherapy Outcomes Using Biomaterials. Angew. Chem. Int. Ed..

[9] Lei K., Tang L. (2019). Surgery-free injectable macroscale biomaterials for local cancer immunotherapy. Biomater. Sci..

[10] Gong Y., Chen M., Tan Y., Shen J., Jin Q., Deng W., Sun J., Wang C., Liu Z., Chen Q. (2020). Injectable Reactive Oxygen Species-Responsive SN38 Prodrug Scaffold with Checkpoint Inhibitors for Combined Chemoimmunotherapy. ACS Appl. Mater. Interfaces.

[11] Neves S.C., Moroni L., Barrias C.C., Granja P.L. (2020). Leveling Up Hydrogels: Hybrid Systems in Tissue Engineering. Trends Biotechnol..

[12] Khosravimelal S., Mobaraki M., Eftekhari S., Ahearne M., Seifalian A.M., Gholipourmalekabadi M. (2021). Hydrogels as Emerging Materials for Cornea Wound Healing. Small.

[13] Zhang F., Li Y.-H., Li J.-Y., Tang Z.-R., Xu Y.-J. (2019). 3D graphene-based gel photocatalysts for environmental pollutants degradation. Environ. Pollut..

[14] Singh A., Qin H., Fernandez I., Wei J., Lin J., Kwak L.W., Roy K. (2011). An injectable synthetic immune-priming center mediates efficient T-cell class switching and T-helper 1 response against B cell lymphoma. J. Control. Release.

[15] Weiden J., Voerman D., Dölen Y., Das R.K., van Duffelen A., Hammink R., Eggermont L., Rowan A.E., Tel J., Figdor C.G. (2018). Injectable Biomimetic Hydrogels as Tools for Efficient T Cell Expansion and Delivery. Front. Immunol..

[16] Kharkar P.M., Kiick K.L., Kloxin A.M. (2013). Designing degradable hydrogels for orthogonal control of cell microenvironments. Chem. Soc. Rev..

[17] Yu S., He C., Chen X. (2018). Injectable Hydrogels as Unique Platforms for Local Chemotherapeutics-Based Combination Antitumor Therapy. Macromol. Biosci..

[18] Wang Y., Wang Z., Wu K., Wu J., Meng G., Liu Z., Guo X. (2017). Synthesis of cellulose-based double-network hydrogels demonstrating high strength, self-healing, and antibacterial properties. Carbohydr. Polym..

[19] Koshy S.T., Ferrante T.C., Lewin S.A., Mooney D.J. (2014). Injectable, porous, and cell-responsive gelatin cryogels. Biomaterials.

[20] Leach D.G., Young S., Hartgerink J.D. (2019). Advances in immunotherapy delivery from implantable and injectable biomaterials. Acta Biomater..

[21] Ishihara M., Kishimoto S., Nakamura S., Sato Y., Hattori H. (2019). Polyelectrolyte Complexes of Natural Polymers and Their Biomedical Applications. Polymers.

[22] Pan J., Jin Y., Lai S., Shi L., Fan W., Shen Y. (2019). An antibacterial hydrogel with desirable mechanical, self-healing and recyclable properties based on triple-physical crosslinking. Chem. Eng. J..

[23] Guo J., Sun H., Lei W., Tang Y., Hong S., Yang H., Tay F., Huang C. (2019). MMP-8-Responsive Polyethylene Glycol Hydrogel for Intraoral Drug Delivery. J. Dent. Res..

[24] Stevens M.M., George J.H. (2005). Exploring and Engineering the Cell Surface Interface. Science.

[25] Wu X., He C., Wu Y., Chen X. (2016). Synergistic therapeutic effects of Schiff’s base cross-linked injectable hydrogels for local co-delivery of metformin and 5-fluorouracil in a mouse colon carcinoma model. Biomaterials.

[26] Liang X., Li L., Li X., He T., Gong S., Zhu S., Zhang M., Wu Q., Gong C. (2021). A spontaneous multifunctional hydrogel vaccine amplifies the innate immune response to launch a powerful antitumor adaptive immune response. Theranostics.

[27] Xu Q., Guo L., Sigen S., Gao Y., Zhou D., Greiser U., Creagh-Flynn J., Zhang H., Dong Y., Cutlar L. (2018). Injectable hyperbranched poly(β-amino ester) hydrogels with on-demand degradation profiles to match wound healing processes. Chem. Sci..

[28] Yu H., Wang Y., Yang H., Peng K., Zhang X. (2017). Injectable self-healing hydrogels formed via thiol/disulfide exchange of thiol functionalized F127 and dithiolane modified PEG. J. Mater. Chem. B.

[29] Kaixuan R., He C., Zhang Z., Ren K., Chen X. (2016). Injectable, Biomolecule-Responsive Polypeptide Hydrogels for Cell Encapsulation and Facile Cell Recovery through Triggered Degradation. ACS Appl. Mater. Interfaces.

[30] Nada A.A., Ali E., Soliman A.A. (2019). Biocompatible chitosan-based hydrogel with tunable mechanical and physical properties formed at body temperature. Int. J. Biol. Macromol..

[31] Yu S., Wang C., Yu J., Wang J., Lu Y., Zhang Y., Zhang X., Hu Q., Sun W., He C. (2018). Injectable Bioresponsive Gel Depot for Enhanced Immune Checkpoint Blockade. Adv. Mater..

[32] Huebsch N., Kearney C., Zhao X., Kim J., Cezar C.A., Suo Z., Mooney D.J. (2014). Ultrasound-triggered disruption and self-healing of reversibly cross-linked hydrogels for drug delivery and enhanced chemotherapy. Proc. Natl. Acad. Sci. USA.

[33] Chao Y., Xu L., Liang C., Feng L., Xu J., Dong Z., Tian L., Yi X., Yang K., Liu Z. (2018). Combined local immunostimulatory radioisotope therapy and systemic immune checkpoint blockade imparts potent antitumour responses. Nat. Biomed. Eng..

[34] Moon H.J., Ko D.Y., Park M.H., Joo M.K., Jeong B. (2012). Temperature-responsive compounds as in situ gelling biomedical materials. Chem. Soc. Rev..

[35] Chen X., Wang M., Yang X., Wang Y., Yu L., Sun J., Ding J. (2019). Injectable hydrogels for the sustained delivery of a HER2-targeted antibody for preventing local relapse of HER2+ breast cancer after breast-conserving surgery. Theranostics.

[36] Phan V.G., Duong H.T.T., Thambi T., Nguyen T.L., Turabee H., Yin Y., Kim S.H., Kim J., Jeong J.H., Lee D.S. (2019). Modularly engineered injectable hybrid hydrogels based on protein-polymer network as potent immunologic adjuvant in vivo. Biomaterials.

[37] Yang F., Shi K., Jia Y., Hao Y., Peng J., Yuan L., Chen Y., Pan M., Qian Z. (2020). A biodegradable thermosensitive hydrogel vaccine for cancer immunotherapy. Appl. Mater. Today.

[38] Van Der Burg S.H., Arens R., Ossendorp F., van Hall T., Melief C.J.M. (2016). Vaccines for established cancer: Overcoming the challenges posed by immune evasion. Nat. Rev. Cancer.

[39] Parry R.V., Chemnitz J.M., Frauwirth K.A., Lanfranco A.R., Braunstein I., Kobayashi S.V., Linsley P.S., Thompson C.B., Riley J.L. (2005). CTLA-4 and PD-1 Receptors Inhibit T-Cell Activation by Distinct Mechanisms. Mol. Cell. Biol..

[40] Iwai Y., Ishida M., Tanaka Y., Okazaki T., Honjo T., Minato N. (2002). Involvement of PD-L1 on tumor cells in the escape from host immune system and tumor immunotherapy by PD-L1 blockade. Proc. Natl. Acad. Sci. USA.

[41] Wang F., Yang S., Palmer N., Fox K., Kohane I.S., Liao K.P., Yu K.-H., Kou S.C. (2021). Real-world data analyses unveiled the immune-related adverse effects of immune checkpoint inhibitors across cancer types. NPJ Precis. Oncol..

[42] Chen M., Tan Y., Dong Z., Lu J., Han X., Jin Q., Zhu W., Shen J., Cheng L., Liu Z. (2020). Injectable Anti-inflammatory Nanofiber Hydrogel to Achieve Systemic Immunotherapy Post Local Administration. Nano Lett..

[43] Chung C.K., Fransen M.F., van der Maaden K., Campos Y., García-Couce J., Kralisch D., Chan A., Ossendorp F., Cruz L.J. (2020). Thermosensitive hydrogels as sustained drug delivery system for CTLA-4 checkpoint blocking antibodies. J. Control. Release.

[44] Rochette L., Guenancia C., Gudjoncik A., Hachet O., Zeller M., Cottin Y., Vergely C. (2015). Anthracyclines/trastuzumab: New aspects of cardiotoxicity and molecular mechanisms. Trends Pharmacol. Sci..

[45] Tolaney S. (2014). New HER2-Positive Targeting Agents in Clinical Practice. Curr. Oncol. Rep..

[46] Lo Y.-W., Sheu M.-T., Chiang W.-H., Chiu Y.-L., Tu C.-M., Wang W.-Y., Wu M.-H., Wang Y.-C., Lu M., Ho H.-O. (2019). In situ chemically crosslinked injectable hydrogels for the subcutaneous delivery of trastuzumab to treat breast cancer. Acta Biomater..

[47] He C., Tang Z., Tian H., Chen X. (2016). Co-delivery of chemotherapeutics and proteins for synergistic therapy. Adv. Drug Deliv. Rev..

[48] Fenton O.S., Tibbitt M.W., Appel E.A., Jhunjhunwala S., Webber M.J., Langer R. (2019). Injectable Polymer–Nanoparticle Hydrogels for Local Immune Cell Recruitment. Biomacromolecules.

[49] Ueda K., Akiba J., Ogasawara S., Todoroki K., Nakayama M., Sumi A., Kusano H., Sanada S., Suekane S., Xu K. (2016). Growth inhibitory effect of an injectable hyaluronic acid–tyramine hydrogels incorporating human natural interferon-α and sorafenib on renal cell carcinoma cells. Acta Biomater..

[50] Robert C. (2020). A decade of immune-checkpoint inhibitors in cancer therapy. Nat. Commun..

[51] Johnson C.N., Pattanayek R., Potet F., Rebbeck R.T., Blackwell D.J., Nikolaienko R., Sequeira V., Le Meur R., Radwański P.B., Davis J.P. (2019). The CaMKII inhibitor KN93-calmodulin interaction and implications for calmodulin tuning of NaV1.5 and RyR2 function. Cell Calcium.

[52] Dai X., Meng J., Deng S., Zhang L., Wan C., Lu L., Huang J., Hu Y., Zhang Z., Li Y. (2020). Targeting CAMKII to reprogram tumor-associated macrophages and inhibit tumor cells for cancer immunotherapy with an injectable hybrid peptide hydrogel. Theranostics.

[53] Burdette D.L., Monroe K.M., Troha K., Iwig J.S., Eckert B., Hyodo M., Hayakawa Y., Vance R.E. (2011). STING is a direct innate immune sensor of cyclic di-GMP. Nature.

[54] Leach D.G., Dharmaraj N., Piotrowski S.L., Lopez-Silva T.L., Lei Y., Sikora A.G., Young S., Hartgerink J.D. (2018). STINGel: Controlled release of a cyclic dinucleotide for enhanced cancer immunotherapy. Biomaterials.

[55] Yang P., Song H., Qin Y., Huang P., Zhang C., Kong D., Wang W. (2018). Engineering Dendritic-Cell-Based Vaccines and PD-1 Blockade in Self-Assembled Peptide Nanofibrous Hydrogel to Amplify Antitumor T-Cell Immunity. Nano Lett..

[56] Song H., Huang P., Niu J., Shi G., Zhang C., Kong D., Wang W. (2018). Injectable polypeptide hydrogel for dual-delivery of antigen and TLR3 agonist to modulate dendritic cells in vivo and enhance potent cytotoxic T-lymphocyte response against melanoma. Biomaterials.

[57] Yin Y., Li X., Ma H., Zhang J., Yu D., Zhao R., Yu S., Nie G., Wang H. (2021). In Situ Transforming RNA Nanovaccines from Polyethylenimine Functionalized Graphene Oxide Hydrogel for Durable Cancer Immunotherapy. Nano Lett..

[58] Koh J., Kim S., Lee S.N., Kim S.-Y., Kim J.-E., Lee K.Y., Kim M.S., Heo J.Y., Park Y.M., Ku B.M. (2021). Therapeutic efficacy of cancer vaccine adjuvanted with nanoemulsion loaded with TLR7/8 agonist in lung cancer model. Nanomed. Nanotechnol. Biol. Med..

[59] Sun Z., Liang J., Dong X., Wang C., Kong D., Lv F. (2018). Injectable Hydrogels Coencapsulating Granulocyte-Macrophage Colony-Stimulating Factor and Ovalbumin Nanoparticles to Enhance Antigen Uptake Efficiency. ACS Appl. Mater. Interfaces.

[60] Song H., Yang P., Huang P., Zhang C., Kong D., Wang W. (2019). Injectable polypeptide hydrogel-based co-delivery of vaccine and immune checkpoint inhibitors improves tumor immunotherapy. Theranostics.

[61] Battaglini E., Goldstein D., Grimison P., McCullough S., Mendoza-Jones P., Park S.B. (2021). Chemotherapy-Induced Peripheral Neurotoxicity in Cancer Survivors: Predictors of Long-Term Patient Outcomes. J. Natl. Compr. Cancer Netw..

[62] Zhao R., Liu X., Yang X., Jin B., Shao C., Kang W., Tang R. (2018). Nanomaterial-Based Organelles Protect Normal Cells against Chemotherapy-Induced Cytotoxicity. Adv. Mater..

[63] Pozzi C., Cuomo A., Spadoni I., Magni E., Silvola A., Conte A., Sigismund S., Ravenda P.S., Bonaldi T., Zampino M.G. (2016). The EGFR-specific antibody cetuximab combined with chemotherapy triggers immunogenic cell death. Nat. Med..

[64] Ren X., Wang N., Zhou Y., Song A., Jin G., Li Z., Luan Y. (2021). An injectable hydrogel using an immunomodulating gelator for amplified tumor immunotherapy by blocking the arginase pathway. Acta Biomater..

[65] Liu M., Cao Z., Zhang R., Chen Y., Yang X. (2021). Injectable Supramolecular Hydrogel for Locoregional Immune Checkpoint Blockade and Enhanced Cancer Chemo-Immunotherapy. ACS Appl. Mater. Interfaces.

[66] Dong X., Yang A., Bai Y., Kong D., Lv F. (2020). Dual fluorescence imaging-guided programmed delivery of doxorubicin and CpG nanoparticles to modulate tumor microenvironment for effective chemo-immunotherapy. Biomaterials.

[67] Lv Q., He C., Quan F., Yu S., Chen X. (2018). DOX/IL-2/IFN-γ co-loaded thermo-sensitive polypeptide hydrogel for efficient melanoma treatment. Bioact. Mater..

[68] Liu L.F., Desai S.D., Li T.-K., Mao Y., Sun M., Sim S.-P. (2000). Mechanism of Action of Camptothecin. Ann. N. Y. Acad. Sci..

[69] Wang F., Xu D., Su H., Zhang W., Sun X., Monroe M.K., Chakroun R.W., Wang Z., Dai W., Oh R. (2020). Supramolecular prodrug hydrogelator as an immune booster for checkpoint blocker–based immunotherapy. Sci. Adv..

[70] Wang F., Su H., Xu D., Dai W., Zhang W., Wang Z., Anderson C.F., Zheng M., Oh R., Wan F. (2020). Tumour sensitization via the extended intratumoural release of a STING agonist and camptothecin from a self-assembled hydrogel. Nat. Biomed. Eng..

[71] Wang C., Wang J., Zhang X., Yu S., Wen D., Hu Q., Ye Y., Bomba H., Hu X., Liu Z. (2018). In situ formed reactive oxygen species–responsive scaffold with gemcitabine and checkpoint inhibitor for combination therapy. Sci. Transl. Med..

[72] Ruan H., Hu Q., Wen D., Chen Q., Chen G., Lu Y., Wang J., Cheng H., Lu W., Gu Z. (2019). A Dual-Bioresponsive Drug-Delivery Depot for Combination of Epigenetic Modulation and Immune Checkpoint Blockade. Adv. Mater..

[73] Hu Z.I., Ho A.Y., McArthur H.L. (2017). Combined Radiation Therapy and Immune Checkpoint Blockade Therapy for Breast Cancer. Int. J. Radiat. Oncol. Biol. Phys..

[74] Morris Z.S., Guy E.I., Francis D.M., Gressett M.M., Werner L., Carmichael L.L., Yang R., Armstrong E.A., Huang S., Navid F. (2016). In Situ Tumor Vaccination by Combining Local Radiation and Tumor-Specific Antibody or Immunocytokine Treatments. Cancer Res..

[75] Liu Q., Zhang D., Qian H., Chu Y., Yang Y., Shao J., Xu Q., Liu B. (2020). Superior Antitumor Efficacy of IFN-α2b-Incorporated Photo-Cross-Linked Hydrogels Combined with T Cell Transfer and Low-Dose Irradiation Against Gastric Cancer. Int. J. Nanomed..

[76] Li J., Rao J., Pu K. (2018). Recent progress on semiconducting polymer nanoparticles for molecular imaging and cancer phototherapy. Biomaterials.

[77] Sun Q., Yang Z., Lin M., Peng Y., Wang R., Du Y., Zhou Y., Li J., Qi X. (2021). Phototherapy and anti-GITR antibody-based therapy synergistically reinvigorate immunogenic cell death and reject established cancers. Biomaterials.

[78] Yao Q., Lan Q.-H., Jiang X., Du C.-C., Zhai Y.-Y., Shen X., Xu H.-L., Xiao J., Kou L., Zhao Y.-Z. (2020). Bioinspired biliverdin/silk fibroin hydrogel for antiglioma photothermal therapy and wound healing. Theranostics.

[79] Wang H., Jiang L., Wu H., Zheng W., Kan D., Cheng R., Yan J., Yu C., Sun S.-K. (2019). Biocompatible Iodine–Starch–Alginate Hydrogel for Tumor Photothermal Therapy. ACS Biomater. Sci. Eng..

[80] Kobayashi H., Choyke P.L. (2019). Near-Infrared Photoimmunotherapy of Cancer. Acc. Chem. Res..

[81] Ng C.W., Li J., Pu K. (2018). Recent Progresses in Phototherapy-Synergized Cancer Immunotherapy. Adv. Funct. Mater..

[82] Song C., Phuengkham H., Kim Y.S., Dinh V.V., Lee I., Shin I.W., Shin H.S., Jin S.M., Um S.H., Lee H. (2019). Syringeable immunotherapeutic nanogel reshapes tumor microenvironment and prevents tumor metastasis and recurrence. Nat. Commun..

[83] Chen Q., Wang C., Zhang X., Chen G., Hu Q., Li H., Wang J., Wen D., Zhang Y., Lu Y. (2019). In situ sprayed bioresponsive immunotherapeutic gel for post-surgical cancer treatment. Nat. Nanotechnol..

[84] Huang X., Lu Y., Guo M., Du S., Han N. (2021). Recent strategies for nano-based PTT combined with immunotherapy: From a biomaterial point of view. Theranostics.

[85] Jia Y.P., Shi K., Yang F., Liao J.F., Han R.X., Yuan L.P., Hao Y., Pan M., Xiao Y., Qian Z.Y. Multifunctional Nanoparticle Loaded Injectable Thermoresponsive Hydrogel as NIR Controlled Release Platform for Local Photothermal Immunotherapy to Prevent Breast Cancer Postoperative Recurrence and Metastases. https://onlinelibrary.wiley.com/doi/epdf/10.1002/adfm.202001059.

[86] Wu Y., Li Q., Shim G., Oh Y.-K. (2021). Melanin-loaded CpG DNA hydrogel for modulation of tumor immune microenvironment. J. Control. Release.

[87] Ye X., Liang X., Chen Q., Miao Q., Chen X., Zhang X., Mei L. (2019). Surgical Tumor-Derived Personalized Photothermal Vaccine Formulation for Cancer Immunotherapy. ACS Nano.

[88] Huang L., Li Y., Du Y., Zhang Y., Wang X., Ding Y., Yang X., Meng F., Tu J., Luo L. (2019). Mild photothermal therapy potentiates anti-PD-L1 treatment for immunologically cold tumors via an all-in-one and all-in-control strategy. Nat. Commun..

[89] Hou X.-L., Dai X., Yang J., Zhang B., Zhao D.-H., Li C.-Q., Yin Z.-Y., Zhao Y.-D., Liu B. (2020). Injectable polypeptide-engineered hydrogel depot for amplifying the anti-tumor immune effect induced by chemo-photothermal therapy. J. Mater. Chem. B.

[90] Fei Z., Fan Q., Dai H., Zhou X., Xu J., Ma Q., Maruyama A., Wang C. (2021). Physiologically triggered injectable red blood cell-based gel for tumor photoablation and enhanced cancer immunotherapy. Biomaterials.

[91] Yu X., Zheng H., Chan M.T.V., Wu W.K.K. (2018). Immune consequences induced by photodynamic therapy in non-melanoma skin cancers: A review. Environ. Sci. Pollut. Res..

[92] Hwang H.S., Shin H., Han J., Na K. (2018). Combination of photodynamic therapy (PDT) and anti-tumor immunity in cancer therapy. J. Pharm. Investig..

[93] Meng Z., Zhou X., Xu J., Han X., Dong Z., Wang H., Zhang Y., She J., Xu L., Wang C. (2019). Light-Triggered In Situ Gelation to Enable Robust Photodynamic-Immunotherapy by Repeated Stimulations. Adv. Mater..

[94] Shu G., Zhu W., Jiang Y., Li X., Pan J., Zhang X., Zhang X., Sun S. (2021). Persistent Luminescence Immune Hydrogel for Photodynamic-Immunotherapy of Tumors In Vivo. Adv. Funct. Mater..

